# Molecular Signature of Cisplatin Resistance in Ovarian Cancer Identifies Therapeutic Opportunities for Re-sensitization

**DOI:** 10.7150/jca.124252

**Published:** 2026-01-01

**Authors:** Mahmoud Mansour, Sabrina van Ginkel, Maaz Alata, Ibrahim Bani, Isra Elhussin

**Affiliations:** 1 Benjamin-Carver FIRST Scholar (UAB/TU Partnership), Biology Department, Center for Biomedical Research (CBR), Tuskegee University, Tuskegee, AL, USA.; 2 Department of Anatomy, Physiology and Pharmacology, Auburn University, Auburn, AL, USA.; 3 Department of Internal Medicine, Section of Medical Oncology, Security Forces Hospital, Riyadh, Saudi Arabia.; 4 Department of Community Medicine, Faculty of Medicine and Surgery, National University, Sudan.; 5 Chronic Disease Epidemiology Department, Yale School of Public Health, New Haven, CT, USA.

**Keywords:** Ovarian cancer, cisplatin-resistant, nuclear factor kappa B (NF-κB), ZBTB33 (Kaiso), Therapeutic Opportunities.

## Abstract

Cisplatin remains a standard first-line therapy for epithelial ovarian cancer; however, chemoresistance leads to poor prognosis and high recurrence. Analysis of The Cancer Genome Atlas confirmed improved overall survival in cisplatin-sensitive tumors, underscoring the need for strategies to overcome resistance in clinical settings. Integrative bioinformatics of cisplatin-treated ovarian cancer datasets from the Gene Expression Omnibus (n=255) identified six molecular drivers of resistance: Kaiso (ZBTB33), pregnane X receptor (PXR), NF-κB, HER2 (ERBB2), P-glycoprotein (P-gp/ABCB1), and HIF1A. These targets were validated in ovarian tumor specimens via immunohistochemistry, confirming elevated expression in chemo-resistant disease. Additionally, the quantitative real-time PCR analysis confirms the transcriptional upregulation of the six resistance-associated genes in cisplatin-resistant SKOV3 and OVCAR-5 ovarian cancer cells, consistent with the immunohistochemistry findings. The average fold change in mRNA transcripts ranged from 2.4 for P-glycoprotein to 5 for both NF-kB and Kaiso. Although less well studied in ovarian cancer, Kaiso is known to regulate EMT and tumor invasion in other solid tumors. Functional studies using SKOV3 and OVCAR-5 cell lines demonstrated that knockdown of Kaiso via RNA interference significantly increased cisplatin-induced cell death, indicating a direct role in therapeutic resistance. Furthermore, we investigated the synergistic effects of combining stearidonic acid (SDA), a plant-based omega-3 fatty acid known to inhibit NF-κB, with cisplatin on cell death in SKOV3 and OVCAR-5 cell lines, and compared the results with those of each compound used individually. Interestingly, co-treatment with stearidonic acid (SDA) synergistically enhanced the cytotoxicity of cisplatin at a lower dose in both cell models. These findings reveal a clinically relevant resistance signature and highlight the therapeutic potential of combinatorial strategies that target both transcriptional regulators (e.g., Kaiso) and inflammatory signaling (e.g., NF-κB). Dual targeting of these pathways may resensitize tumors to cisplatin and improve outcomes for patients with advanced ovarian cancer.

## 1. Introduction

Epithelial ovarian cancer (EOC) remains the most lethal gynecologic malignancy worldwide, accounting for an estimated 313,959 new cases and 207,252 deaths annually 1, 2. Despite advances in surgical techniques and chemotherapy regimens, the five-year survival rate for advanced-stage disease remains below 30%, largely due to the high rate of recurrence and chemoresistance 3-5. Standard first-line therapy for EOC includes cytoreductive surgery followed by platinum-based chemotherapy, most commonly using cisplatin or carboplatin in combination with paclitaxel 6, 7. While initial responses are often favorable, most patients relapse within two years and develop resistance to platinum-based therapy, which severely limits treatment options and adversely impacts overall survival 8.

Cisplatin resistance is a complex and multifactorial process involving both intrinsic and acquired mechanisms 9-14. These include enhanced DNA repair, altered drug uptake and efflux, epithelial-to-mesenchymal transition (EMT), activation of survival signaling pathways, evasion of apoptosis, and changes in the tumor microenvironment 15, 16. Furthermore, emerging evidence indicates that transcriptional regulators and stress-responsive pathways such as NF-κB 17, 18 Hypoxia-inducible factors (HIFs) 19, and nuclear receptors 20 contribute to resistance by promoting tumor cell adaptation to chemotherapy-induced stress 21. Despite numerous preclinical studies and clinical trials, effective strategies to reverse or overcome platinum resistance remain elusive, highlighting a critical unmet need in ovarian cancer therapy 5.

Transcriptomic profiling and integrative bioinformatics analyses have become valuable tools in identifying molecular signatures associated with drug resistance. By analyzing gene expression data from cisplatin-treated tumors, researchers can uncover key pathways and potential therapeutic targets. Targeting master regulators of chemoresistance, such as transcription factors and nuclear receptors, may offer a more durable approach than inhibiting single downstream effectors. Additionally, combining chemotherapeutics with natural compounds that modulate inflammatory or metabolic pathways has shown promise in preclinical models, potentially enhancing drug efficacy while minimizing toxicity.

In this study, we conducted a secondary bioinformatics analysis of publicly available transcriptomic data from cisplatin-treated ovarian cancer patients to identify a resistance-associated gene signature. We identified six key regulators—ZBTB33 (Kaiso), NF-κB, pregnane X receptor (PXR), HER2, P-glycoprotein (ABCB1), and HIF-1α—that were significantly upregulated in resistant tumors. These genes are involved in critical processes such as transcriptional repression, inflammatory signaling, xenobiotic metabolism, hypoxia adaptation, and drug efflux. To functionally validate these findings, we evaluated the effects of stearidonic acid (SDA), a plant-derived omega-3 fatty acid with known NF-κB inhibitory properties 22, in combination with cisplatin in resistant ovarian cancer cell lines. We further investigated the role of Kaiso in cisplatin-resistance through targeted gene knockdown. Kaiso regulates EMT and metastasis in other tumors 23, 24.

Building on prior findings that the plant-derived omega-3 fatty acid stearidonic acid (SDA) inhibits NF-κB in prostate cancer 25, we examined whether SDA could synergize with cisplatin to overcome chemoresistance in cisplatin-resistant SKOV3 and OVCAR-5 ovarian cancer cells.

## 2. Materials and Methods

### 2.1 Bioinformatics analysis

#### 2.1.1 Analysis of cisplatin chemoresistance using geo microarray datasets

Gene Expression Omnibus (GEO) datasets of cisplatin-treated ovarian cancer patients, GSE47856 (171 samples), GSE23553 (56 samples), and GSE24590 (28 samples) were downloaded from https://www.ncbi.nlm.nih.gov/geo, along with their respective platform (GPL) files. Gene symbols associated with each read were extracted from the GPL files and merged with the GSE data using the merge function in R. These datasets included a total of 255 ovarian cancer patient samples, most of whom received cisplatin-based chemotherapy. Only samples with documented clinical responses to treatment were included in the analysis to investigate cisplatin resistance.

#### 2.1.2 Survival data analysis

Overall survival (OS) probabilities were calculated using the Kaplan-Meier method, with significance assessed via the log-rank test. Clinical and genomic data for 563 ovarian cancer patients were obtained from The Cancer Genome Atlas (TCGA), originally published in Nature 26 and accessed through the cBioPortal for Cancer Genomics (https://www.cbioportal.org/).

Survival curves were generated using R Studio based on survival time (months), survival status, and platinum sensitivity.

#### 2.1.3 Pathway studio analysis

To explore the molecular mechanisms underlying chemoresistance, differentially expressed gene (DEG) lists were imported into Pathway Studio Web 11.1 (Elsevier), a network-based systems biology platform. Subnetwork Enrichment Analysis (SNEA) was conducted to identify significantly enriched signaling and regulatory pathways. Pathways were prioritized based on z-scores, p-values, and biological relevance. The analysis also accounted for tissue-specific expression, directional relationships (e.g., activation or inhibition), and disease associations to generate mechanistic hypotheses.

### 2.2 Immunohistochemistry (IHC)

Formalin-fixed, paraffin-embedded (FFPE) ovarian cancer tissue sections representing stages I-III and corresponding normal tissues (US Biomax Inc., Rockville, MD; cat# OV952) were analyzed for the localization of six proteins identified from the GEO dataset. Sections were deparaffinized in xylene, rehydrated through graded alcohols, and subjected to antigen retrieval using pressure cooking for 10 minutes. Endogenous peroxidase activity was quenched with 3% hydrogen peroxide for 5 minutes. Blocking was performed with 5% normal goat serum and 2% BSA in PBS (pH 7.3) for 1 hour at room temperature in a humidity chamber 25.

Slides were incubated with primary antibodies (Abcam or Santa Cruz) at recommended dilutions, followed by horseradish peroxidase (HRP)-conjugated secondary antibodies (anti-mouse or anti-rabbit) for 40 minutes. Antigen-antibody complexes were visualized with 3,3'-diamino-benzidine tetrahydrochloride (DAB, Sigma-Aldrich, MO) for 7 minutes and counterstained with hematoxylin (Sigma-Aldrich, MO) for 1 minute. Finally, slides were dehydrated through ethanol series, cleared in xylene, and mounted with Paramount medium. Images were captured using a light microscope.

### 2.3 PCR analysis

Total RNA was isolated from SKOV3 and OVCAR5 cell lines using TRI Reagent (Sigma, St. Louis, MO), following the manufacturer's protocol. RNA concentration and purity (260/280 ratio) were determined via UV spectrophotometry (DU6,4,0, Beckman Coulter, Fullerton, CA). Samples with ratios ≥ 1.8 were used for cDNA synthesis using the Superscript III First-Strand cDNA Synthesis Kit (Invitrogen, Grand Island, NY).

Validated All-in-One™ qPCR primers for Kaiso, PXR, NF-κB, HER2, ABCB1 (P-gp), HIF1A, and ACTB (β-actin) were obtained from GeneCopoeia and confirmed for specificity and efficiency. QPCR reactions (25 µL) included 12.5 µL SYBR Green master mix, 1 µL cDNA, 1 µL primer, and 10.5 µL PCR-grade water. PCR was run using a Bio-Rad MyiQ thermal cycler with the following cycling conditions: 95 °C for 15 minutes, followed by 40 cycles of 95 °C (30 sec), 55 °C (30 sec), and 72 °C (30 sec). Data were analyzed using the 2^-ΔΔCt method 27. PCR product identity was verified by melting curve analysis and 2% agarose gel electrophoresis.

### 2.4 Ovarian cancer cell culture

Cisplatin-resistant SKOV-3 and OVCAR-5 ovarian cancer cell lines were purchased from ATCC (Manassas, VA). SKOV-3 cells were cultured in McCoy's 5A medium supplemented with 1.5 mM L-glutamine, 2,2,0,0 mg/L sodium bicarbonate, 10% fetal bovine serum (FBS, Atlanta Biologicals), and 1% penicillin/streptomycin (Sigma). OVCAR-5 cells were cultured in RPMI-1640 medium supplemented with HEPES, L-glutamine, 10% FBS, and 1% penicillin/streptomycin.

### 2.5 MTT cell viability assay

MTT assays were performed using the manufacturer's protocol (ATCC) and our previous methods 25. SKOV3 and OVCAR-5 cells were seeded in 96-well plates (10⁵ cells/well) and incubated overnight. Cells were treated with 5 µM cisplatin or stearidonic acid (SDA) at concentrations of 25, 50, 100, or 200 µM for 48 hours.

After treatment, 10 µL of MTT solution (5 mg/mL) was added to each well and incubated in a CO2 incubator at 37^0^C for 3-4 hours. To solubilize the formazan crystals, 100 µL of SDS-HCl detergent solution (10% SDS in 0.01 N HCl) was added, and plates were incubated for 5-6 hours at room temperature in the dark. Absorbance was measured at 570 nm using a SpectraMax-Plus-384 microplate reader (Molecular Devices). Experiments were conducted in triplicate and repeated three times.

### 2.6 RNA interference

RNA interference was performed as previously described in our laboratory 28. To generate stable short hairpin RNA (shRNA) kaiso cells, the HuSH 29-mer for Kaiso was provided in the pRFP-C-RS plasmid driven by the U6-RNA promoter (OriGene, Rockville, MD). Plasmid DNA pRFP-C-RS, containing the puromycin-resistant gene, expressing Kaiso-specific shRNA, and scrambled shRNA control were transfected into SKOV3 cells using Lipofectamine 2000 (Invitrogen, Waltham, MA). The medium was replaced by T medium containing 2 µg/mL puromycin for selection of antibiotic-resistant colonies over a period of three weeks. The puromycin-resistant cells were further selected using red fluorescence protein as a marker to enrich Kaiso-depleted cells expressing shRNA. Sh-Kaiso cells were plated at clonal densities, and more than 20 clones were chosen to determine the degree of knockdown. Clones with the lowest Kaiso levels were retained for further analysis using PCR, flow cytometry and western blotting.

### 2.7 Western blotting (WB)

Immunoblotting was performed as previously described 29. Briefly, cells were grown in 6-well plates or Petri dishes and lysed in RIPA buffer (Sigma Aldrich, St. Louis, MO) containing a set of protease inhibitors (Sigma, St. Louis, MO). The cell lysates were homogenized then centrifuged at 13000g for 15 min to recover cell supernatant and remove cell debris. Protein concentrations were determined with the Bradford assay. Protein aliquots (30-100 micrograms) in Laemmli sample buffer were heated to 95 °C for five minutes then cooled on ice. Next, the protein was resolved on precast 10% tris-HCl mini polyacrylamide gels (Bio-Rad, Hercules, CA) and separated by SDS PAGE. Electrophoresed proteins were next transferred to polyvinylidene fluoride (PVDF) membrane (Bio-Rad, Hercules, CA). Membranes were blocked with 5% non-fat dry milk in tris-buffered saline (1% Tween-20 buffer (TBST), probed with primary antibodies diluted appropriately in block buffer. Primary antibodies and beta actin used in the study (NF-κB p65 Antibody (A-12): sc-514451 and beta actin antibody (4E8H3): sc-130065) were obtained from Santa Cruz.

Western blots were incubated with appropriate primary antibodies for 24 or 48 hours using constant shaking at 4 °C. Next, blots were washed to remove unbound antibodies before incubation with the appropriate anti-mouse or rabbit secondary antibodies (1/1000 dilution) for 90 min at room temperature. Membranes were then washed and incubated with chemiluminescent developing reagent (Amersham Biosciences, NJ) for two to five minutes. Membranes were sprayed with HyGlo (Denville Scientific, Metuchen, NJ) and exposed to x-ray film.

### 2.8 Statistical analysis

For MTT experiments, statistics were performed with Prism software (GraphPad, La Jolla, CA). An independent Student's t-test was used to determine statistical differences between treatments. The Overall survival (OS) probabilities were calculated using the Kaplan-Meier method, with significance assessed via the log-rank test.

## 3. Results

### 3.1 Integrated molecular and clinical insights into cisplatin resistance in ovarian cancer

#### 3.1.1 Cisplatin sensitivity correlates with improved patient survival

**Figure 1:**
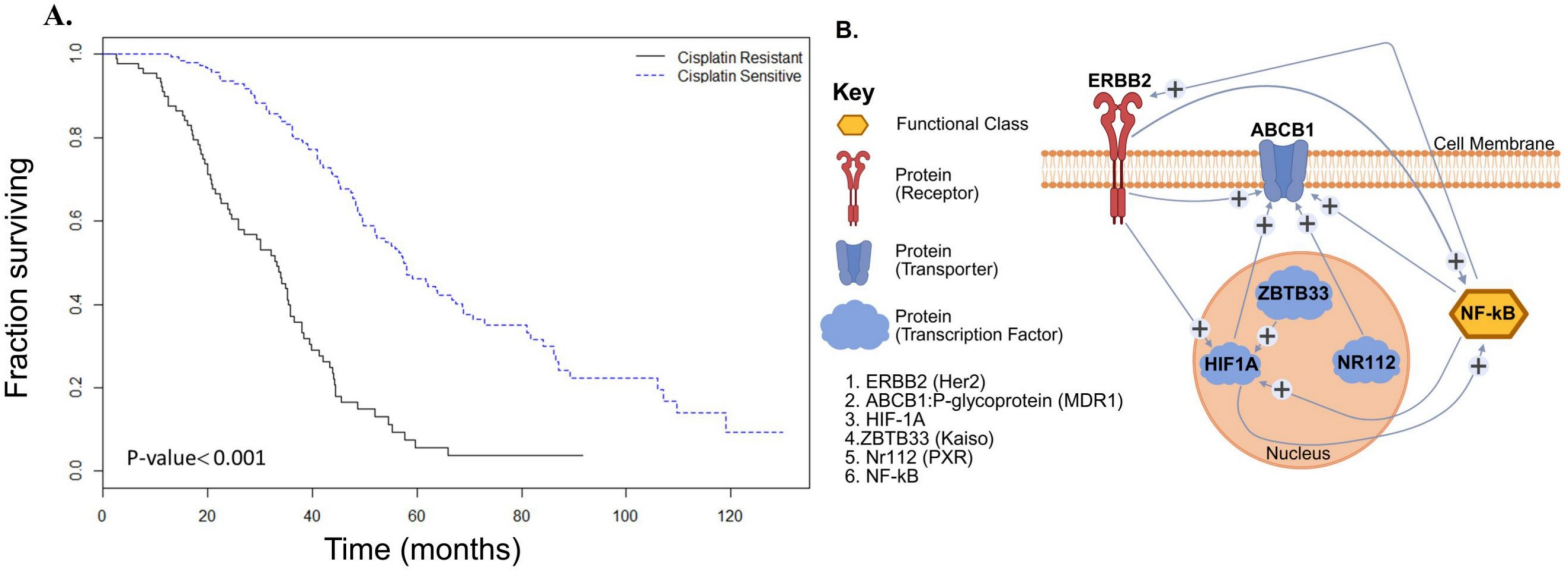
**Integrated molecular and clinical insights into cisplatin resistance in ovarian cancer**. **(A)** Kaplan-Meier survival analysis shows a significant survival advantage in cisplatin-sensitive ovarian cancer patients (dotted line) compared to those with cisplatin-resistant disease (solid line), based on TCGA data (P < 0.001). **(B)** Gene enrichment and interaction network loop generated from microarray analyses of GEO datasets and curated using Pathway Studio Web 11.1, illustrating key regulatory nodes associated with cisplatin resistance. Central signaling molecules include NF-κB, HER2 (ERBB2), P-glycoprotein (ABCB1/MDR1), pregnane X receptor (PXR/NR1I2), ZBTB33 (Kaiso), and HIF1A.

To assess the clinical relevance of cisplatin sensitivity, we performed Kaplan-Meier survival analysis using TCGA data (n = 563). The analysis revealed a significant increase in overall survival in cisplatin-sensitive patients over a 6-year follow-up period (P < 0.001), emphasizing the importance of overcoming resistance (**Figure 1A**).

### 3.2 Bioinformatics analysis identifies six factors associated with cisplatin resistance

Secondary analysis of published GEO microarray data using Pathway Studio identified six key proteins involved in chemoresistance: Kaiso (ZBTB33), HIF1A, NF-κB, HER2 (ERBB2), P-glycoprotein (MDR1/ABCB1), and pregnane X receptor (PXR/NR1I2). Network modeling showed HER2 (ERBB2) and P-gp (ABCB1) as upstream regulators of NF-κB, while Kaiso, HIF1A, and PXR were downstream, suggesting NF-κB as a central node in the resistance pathway. Network modeling showed HER2 (ERBB2) and P-gp (ABCB1) as upstream regulators of NF-κB, while Kaiso, HIF1A, and PXR were downstream, suggesting NF-κB as a central node in the resistance pathway **(Figure 1B)**. This complex gene network centered on NF-κB in epithelial-type ovarian cancers, promotes multidrug resistance (MDR) phenotype through overexpression of ABCB1 (P-gp) as well as interaction with transcriptional regulators and effector molecules, including HER2, PXR, and Kaiso.

### 3.3 Increased expression of resistance-associated proteins in Grade 2 ovarian cancer

**Figure 2:**
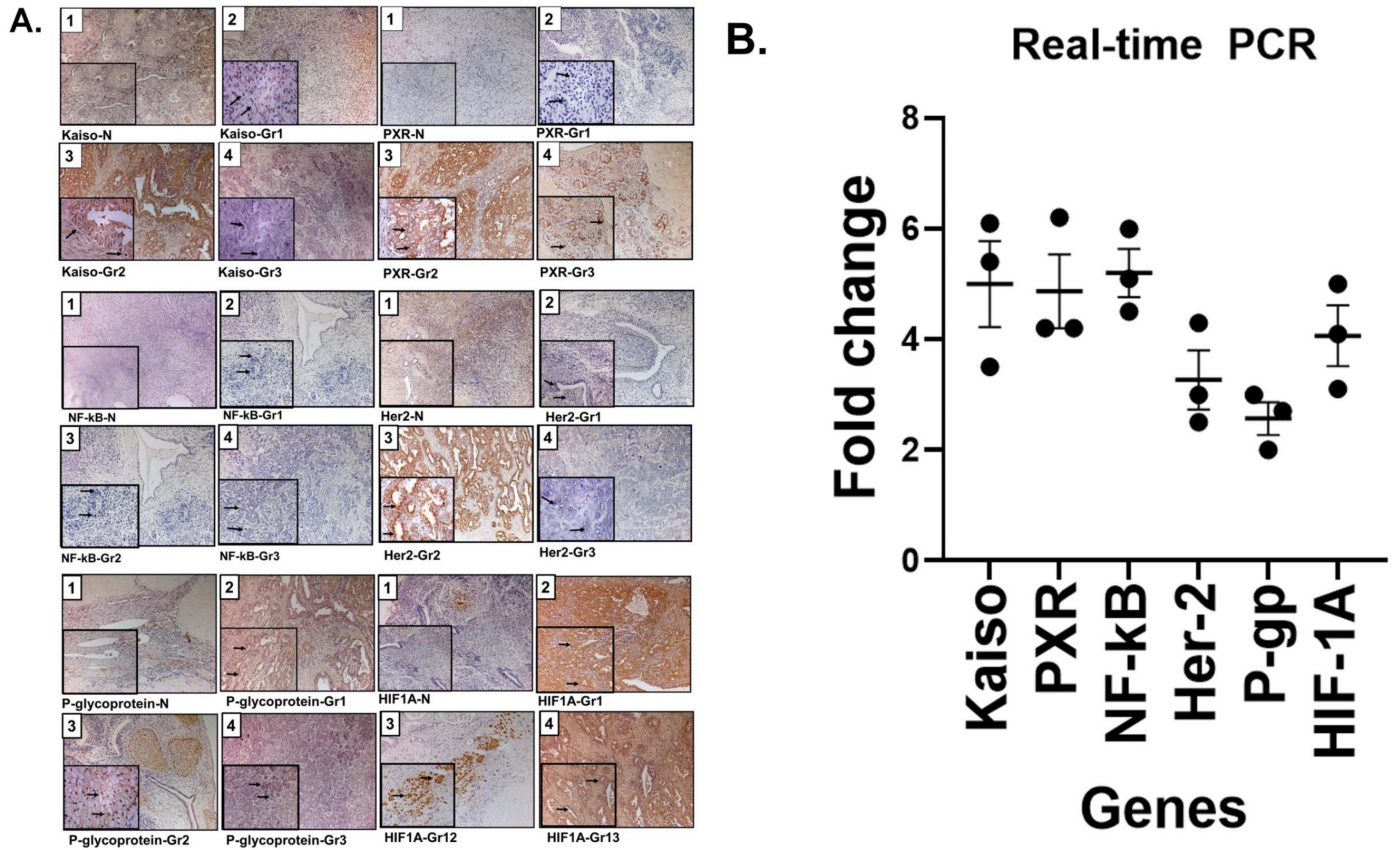
** Validation of cisplatin resistance biomarkers in ovarian cancer: tissue expression and transcript levels in cisplatin-resistant SKOV3 ovarian cancer cell line. (A)** Representative immunohistochemical (IHC) staining of tissue sections from patients with normal ovarian histology (panel 1) and grade 1-3 ovarian cancer (panels 2, 3, and 4), demonstrating expression of nuclear Kaiso, PXR, NF-κB, and HIF1A, as well as membranous HER2 (ERBB2) and P-glycoprotein (P-gp/ABCB1). Grade 2 tumors show markedly increased staining intensity for all six proteins, suggesting upregulation associated with chemoresistance. Insets highlight magnified subcellular localization patterns (nuclear or membranous). These proteins are involved in transcriptional regulation, drug metabolism, hypoxia response, and drug efflux, and were detected using validated antibodies from Abcam/ Santa Cruz Biotechnology. See supplementary table (S1) for antibodies used. **(B)** Quantitative real-time PCR analysis (3 replica) demonstrates transcriptional upregulation of the six resistance-associated genes in the cisplatin-resistant SKOV3 ovarian cancer cell line, corroborating the IHC results. Together, these findings support the clinical relevance of this biomarker panel for stratifying cisplatin-resistant ovarian cancers and informing therapeutic decision-making.

An Immunohistochemistry (IHC) analysis of ovarian tissues revealed elevated expression of all six resistance-associated proteins with more intensity in tumors tissues compared to normal ovarian tissues. We examined NF-κB, Kaiso, PXR, and HER2 across Grades 1-3. Grade 2 tumors showed strong nuclear staining for Kaiso, PXR, NF-κB, and HIF1A, and membrane expression of HER2 and P-gp. These markers showed concordant expression patterns and correlated with moderate tumor differentiation (**Figure 2A**). Notably, protein expression was markedly reduced in Grade 3 tissues.

### 3.4 Quantitative PCR confirms transcriptional upregulation of resistance genes

To validate transcriptional expression, we performed real-time PCR on cisplatin-resistant SKOV3 ovarian cancer cells. All six genes identified by IHC showed significant upregulation, with fold increases ranging from 2.4 for P-gp to 5.0 for both NF-κB and Kaiso (**Figure 2B**). These findings support the protein-level data obtained via IHC as shown in Figure 1B.

### 3.5 Stearidonic acid enhances cisplatin toxicity in resistant ovarian cancer cells

**Figure 3:**
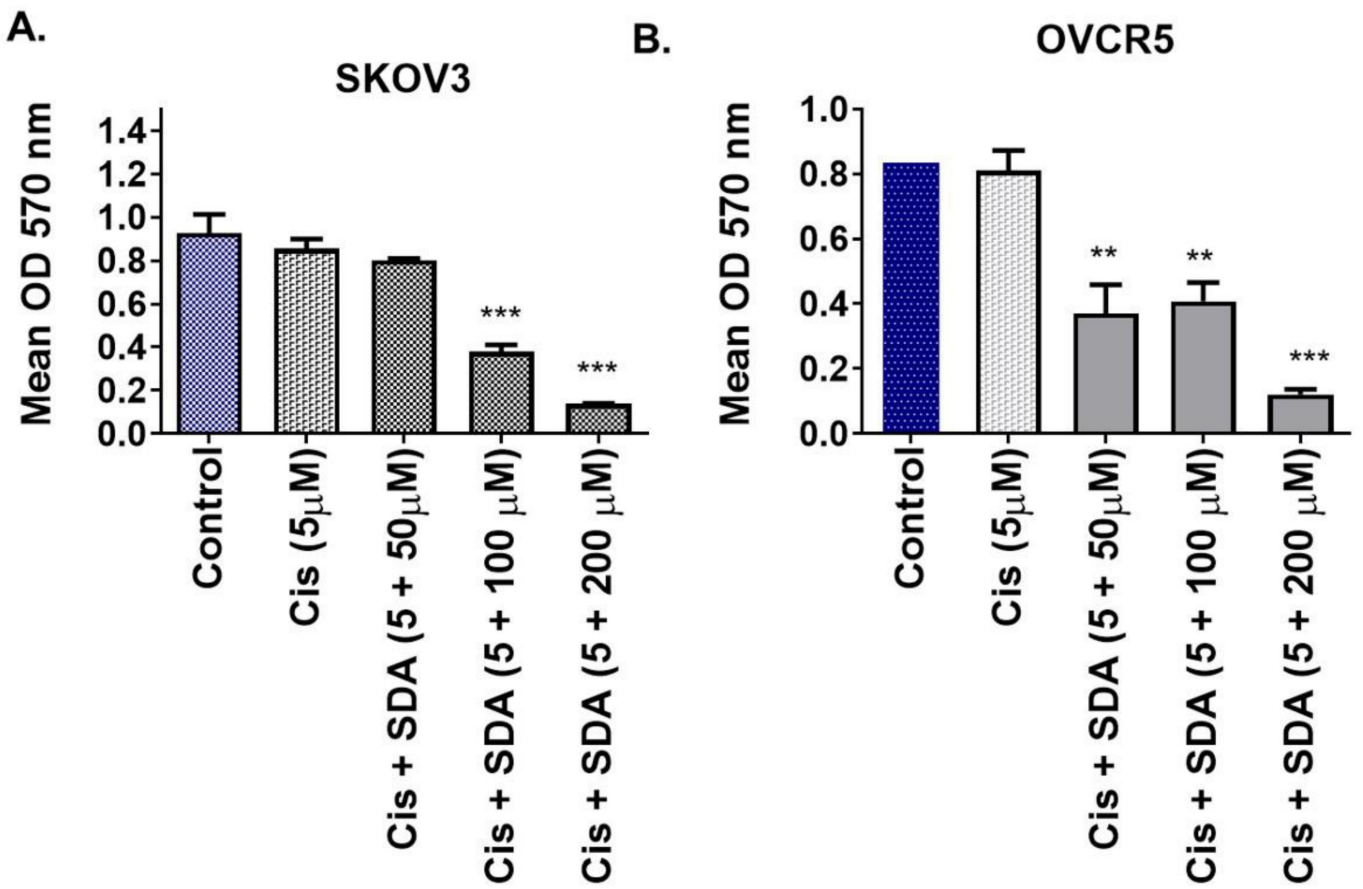
**Stearidonic acid (SDA) enhances cisplatin sensitivity in chemoresistant ovarian cancer cell lines. (A-B)** MTT cell viability assays show that co-treatment with the plant-derived omega-3 fatty acid stearidonic acid (SDA) significantly enhances the cytotoxic efficacy of cisplatin (Cis) in two aggressive, cisplatin-resistant ovarian cancer cell lines. **(A)** In SKOV3 cells, treatment with 5 µM cisplatin alone resulted in modest, non-significant cytotoxicity. However, co-administration with SDA at 100 or 200 µM led to a substantial and significant reduction in cell viability, indicating a synergistic effect.** (B)** Similar trends were observed in OVCAR-5 cells, where even 50 µM SDA significantly potentiated cisplatin-induced cell death. Notably, cisplatin at 5 µM alone did not significantly affect cell viability in either cell line, but its combination with 50-100 µM SDA resulted in marked cancer cell death. These findings suggest that SDA acts as an effective chemosensitizer, capable of restoring cisplatin responsiveness in resistant ovarian cancers, with potential implications for combinatorial therapeutic strategies. See supplementary data (Figure 1) on dose of Cis and SDA selection for this experiment. Experiments were repeated three times.

Building on prior findings that stearidonic acid (SDA) sensitizes prostate cancer cells to taxanes via NF-κB inhibition, we evaluated its effect on cisplatin toxicity in ovarian cancer. Co-treatment of SKOV3 cells with 5 μM cisplatin and 100 μM SDA significantly increased cell death compared to either agent alone (**Figure 3A**). Similar results were observed in OVCAR-5 cells (**Figure 3B**), supporting the synergistic effect of SDA in enhancing cisplatin cytotoxicity.

### 3.6 Kaiso knockdown increases cisplatin sensitivity in SKOV3 ovarian cancer cells

**Figure 4:**
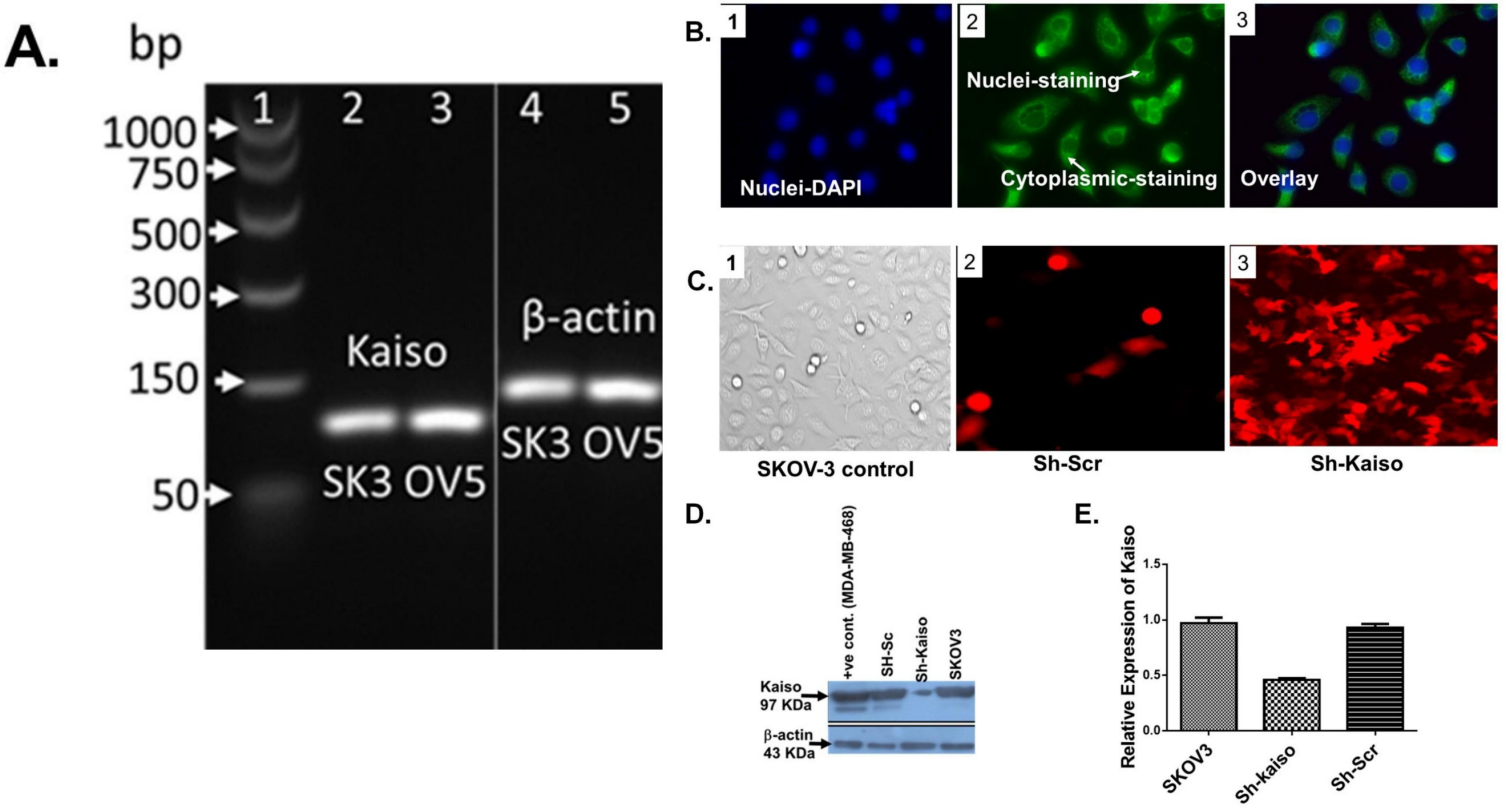
**Kaiso expression and knockdown validation in cisplatin-resistant ovarian cancer cells. (A)** RT-PCR analysis confirms Kaiso mRNA expression in two cisplatin-resistant ovarian cancer cell lines—SKOV3 (SK3) and OVCAR-5 (OV5)—with β-actin used as a housekeeping control. **(B)** Immunofluorescence microscopy of SKOV3 cells (panels 1-3) reveals both nuclear and cytoplasmic localization of Kaiso, consistent with its dual role in transcriptional regulation and cellular signaling. **(C)** Fluorescence imaging demonstrates enrichment of SKOV3 cells transfected with sh-Kaiso (panel 3-RF), labeled with red fluorescent protein (RFP), confirming successful transduction relative to scrambled control (Sh-Scr). **(D)** Western blot analysis further confirms Kaiso protein depletion in Sh-Kaiso-transfected SKOV3 cells (lane 3), with MDA-MB-468 cells and scrambled vector controls serving as positive controls. **(E)** Quantitative real-time PCR analysis validates a significant reduction in Kaiso mRNA levels in sh-Kaiso SKOV3 cells compared to both wild-type and Sh-Scr controls, confirming effective knockdown at the transcript level (see supplementary data (Figure 2) for additional confirmation by Flow cytometry analysis.

**Figure 5:**
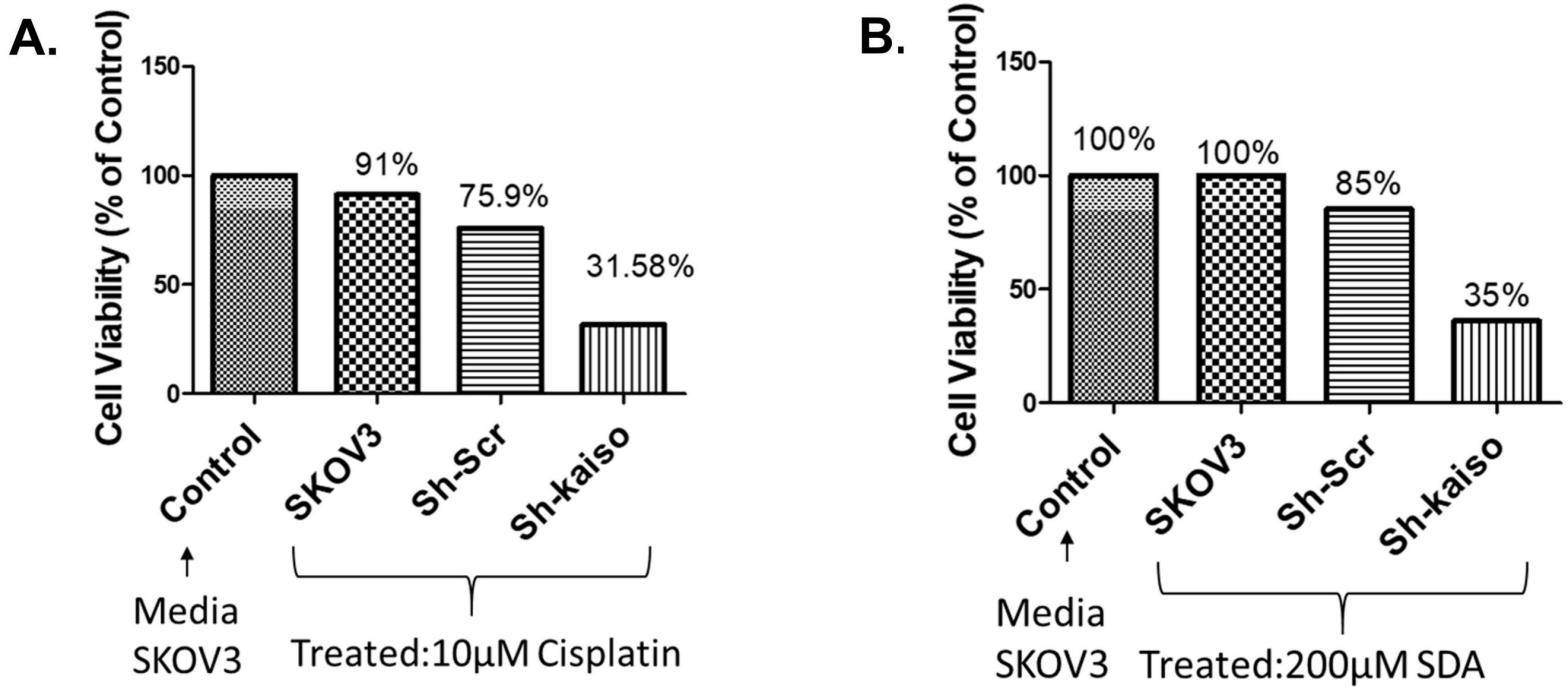
**Kaiso knockdown enhances sensitivity to cisplatin and stearidonic acid (SDA) in cisplatin-resistant SKOV3 cells. (A)** MTT assay shows that SKOV3 cells with Kaiso knockdown exhibit significantly reduced viability (31.6%) following treatment with 10 µM cisplatin, compared to 91% viability in untreated wild-type controls. **(B)** Similarly, Kaiso knockdown cells treated with 200 µM SDA show reduced viability (35%) relative to 100% viability in untreated wild-type controls. These results indicate that Kaiso suppression sensitizes cisplatin-resistant SKOV3 cells to both cisplatin and SDA. Experiment repeated three times.

Previous research has linked nuclear Kaiso expression to tumor aggressiveness and resistance in various cancers 23. To evaluate its role in ovarian cancer, we used RNA interference to knock down Kaiso in SKOV3 cells. PCR and immunofluorescence confirmed Kaiso expression in SKOV3 and OVCAR-5 lines, and its successful silencing (**Figure 4 A-E**). Kaiso knockdown significantly increased the cytotoxic effect of cisplatin and SDA, suggesting its critical role in mediating resistance (**Figure 5A-B**). These results demonstrate that targeting Kaiso, alone or in combination with SDA, can help reverse cisplatin resistance in ovarian cancer.

## 4. Discussion

Epithelial ovarian cancer initially responds well to platinum-based chemotherapy but often recurs with acquired cisplatin resistance, a phenomenon strongly associated with poor prognosis. Most patients relapse within 6-24 months due to drug resistance, leading to significantly reduced overall survival 8^.^ Notably, tumor grade is a key prognostic factor. Patients with low-grade (grade 1) tumors exhibit markedly better median overall survival compared to those with high-grade (grades 2 and 3) tumors—for instance, reported median survival times range from 90.8 months for low-grade tumors to 40.7 months for high-grade tumors 30-32^.^

To address this clinical challenge, we performed a secondary bioinformatics analysis of publicly available transcriptomic data from cisplatin-treated ovarian cancer patients. Using pathway enrichment analysis (Pathway Studio Web 11.1), we systematically identified and prioritized transcriptional networks associated with therapeutic resistance. We then investigated the combinatorial effects of stearidonic acid (SDA) with cisplatin in cisplatin-resistant SKOV3 and OVCAR-5 ovarian cancer cell lines. These preclinical models are widely used to study epithelial-to-mesenchymal transition (EMT), NF-κB signaling, apoptosis evasion, and drug efflux. SKOV3 cells are characterized by p53 deficiency, mesenchymal features, and high migratory capacity, while OVCAR-5 cells exhibit intrinsic resistance to cisplatin and taxanes, with elevated expression of ABC transporters 33.

Kaplan-Meier survival analysis confirmed that patients with cisplatin-sensitive tumors had significantly improved overall survival (Figure 1A), underscoring the clinical relevance of elucidating resistance mechanisms. Our pathway analysis revealed a six-gene resistance signature consisting of ZBTB33 (Kaiso), NF-κB, pregnane X receptor (PXR/NR1I2), HER2 (ERBB2), P-glycoprotein (ABCB1/MDR1), and HIF1A (Figure 1B/Figure 2A and B). These genes regulate inflammatory signaling, transcription, drug efflux, and hypoxia adaptation. We validated their overexpression in ovarian tissue samples ranging from normal to grade I-III tumors, with consistent expression in grade II tumors, suggesting early involvement in resistance 5, 34-36. MTT assays demonstrated that SDA significantly enhanced cisplatin cytotoxicity in both cell lines (Figure 3 A and B). Furthermore, shRNA-mediated knockdown of Kaiso in SKOV3 cells sensitized them to cisplatin, indicating its critical role in chemoresistance 9 (Figure 4).

**ZBTB33 (Kaiso)** is a transcriptional repressor from the POZ-ZF family, with a context-dependent role in cancer. It can function as a tumor suppressor or oncogene depending on its interaction with sequence-specific or methylated DNA targets 37-40. Previous studies show nuclear Kaiso enrichment in aggressive prostate cancers, where its inhibition reduced invasion and metastasis 28, 41, as well as in breast cancers, particularly HER2-driven and triple-negative subtypes 42. Conflicting reports on Kaiso's role in cell proliferation 43 highlight its complex biology. Our findings demonstrate that Kaiso knockdown (Figure 4) restores cisplatin sensitivity in SKOV3 cells (Figure 5), suggesting a functional role in intermediate-grade tumors where resistance emerges early.

**Pregnane X Receptor (PXR)** is a nuclear receptor that regulates xenobiotic metabolism and clearance, including chemotherapeutic agents 44, 45. In ovarian cancer, PXR contributes to resistance by up-regulating drug-metabolizing enzymes and efflux transporters 46, 47. Our data show elevated PXR expression in resistant tumors, supporting its role as a resistance amplifier.

**NF-κB** is a well-established driver of tumor progression and resistance, promoting cell survival, proliferation, metastasis, and immune evasion 17, 48. It also contributes to cancer stem cell maintenance and relapse 49-51. We previously demonstrated that SDA inhibits NF-κB signaling 22 and in this study, SDA effectively sensitized SKOV3 and OVCAR-5 cells to cisplatin (Figure 3).

**HER2 (ERBB2)** is overexpressed in subsets of ovarian cancers, particularly high-grade serous and mucinous histologies, contributing to aggressive behavior 52-54. Although HER2-targeted therapies have shown limited efficacy in unselected ovarian cancer populations, our findings suggest that biomarker-guided strategies that include combination therapy may enhance therapeutic outcomes in resistant cases 55, 56.

**ABCB1 (MDR1)** encodes P-glycoprotein, a drug efflux transporter that reduces intracellular concentrations of chemotherapy agents, mediating multidrug resistance 57, 58. Its overexpression in our cisplatin-resistant cancer cell and tissue samples supports its role as a downstream effector of NF-κB and PXR signaling 59.

**HIF-1α** mediates hypoxia-induced transcription and is implicated in tumor growth, metastasis, and treatment resistance. Its overexpression in ovarian cancer correlates with poor prognosis, making it a promising therapeutic target 19.

While each of these pathways contribute to chemoresistance, single-agent inhibitors have had limited clinical impact due to pathway redundancy and compensatory mechanisms. Our data supports a multi-targeted approach that disrupts the coordinated signaling network sustaining resistance. SDA, a plant-derived omega-3 fatty acid, synergistically enhanced cisplatin-induced cytotoxicity in SKOV3 cells. Previous studies from our group showed that SDA potentiates docetaxel and doxorubicin in prostate cancer models 22, 25. Here, 5 µM cisplatin alone produced minimal cell death, but its combination with 100 µM SDA significantly increased cytotoxicity.

In summary, our integrative transcriptomic analysis and functional experiments suggest that early-stage resistance in ovarian tumors arises from convergence on inflammatory, hypoxic, and xenobiotic-response pathways. Key transcriptional regulators like NF-κB and Kaiso appear to orchestrate these mechanisms. Inhibiting these drivers, either genetically or pharmacologically, may enable precision treatment strategies to overcome resistance before clinical relapses. Our findings support targeting multiple resistance nodes rather than single effectors and provide preclinical evidence that combining SDA with cisplatin may restore chemosensitivity and improve therapeutic outcomes in aggressive ovarian cancer.

## Supplementary Material

Supplementary figures and tables.
