# Supplementary material for: Molecular Signature of Cisplatin Resistance in Ovarian Cancer Identifies Therapeutic Opportunities for Re-sensitization
Source: J Cancer. 2026 Jan 1;17(1):49–58. doi: 10.7150/jca.124252 (PMC12719565; doi:10.7150/jca.124252)
Supplement: Supplementary file 1 — Supplementary figures and tables. [file jcav17p0049s1.pdf]

A. **Cisplatin IC50**

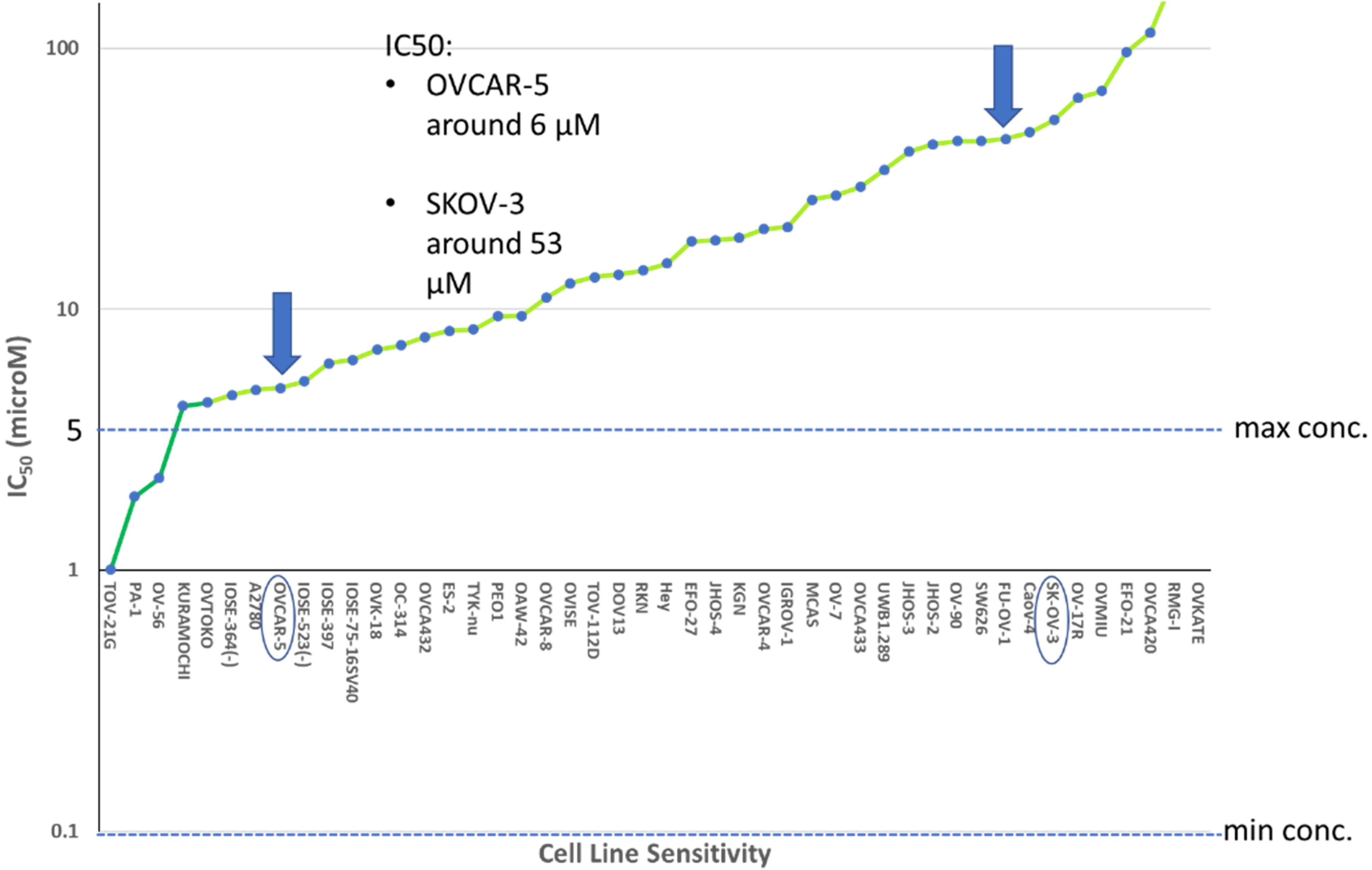

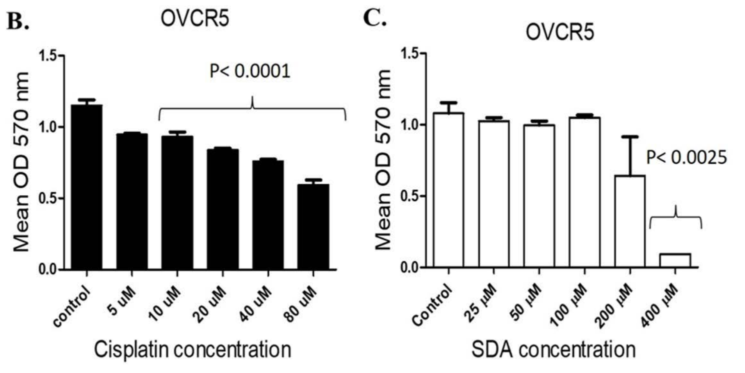
B.
OVCR5
P< 0.0001
Mean OD 570 nm
1.5
1.0
0.5
0.0
control
5 uM
10 uM
20 uM
40 uM
80 uM
Cisplatin concentration
C.
OVCR5
P< 0.0025
Mean OD 570 nm
1.5
1.0
0.5
0.0
Control
25 μM
50 μM
100 μM
200 μM
400 μM
SDA concentration

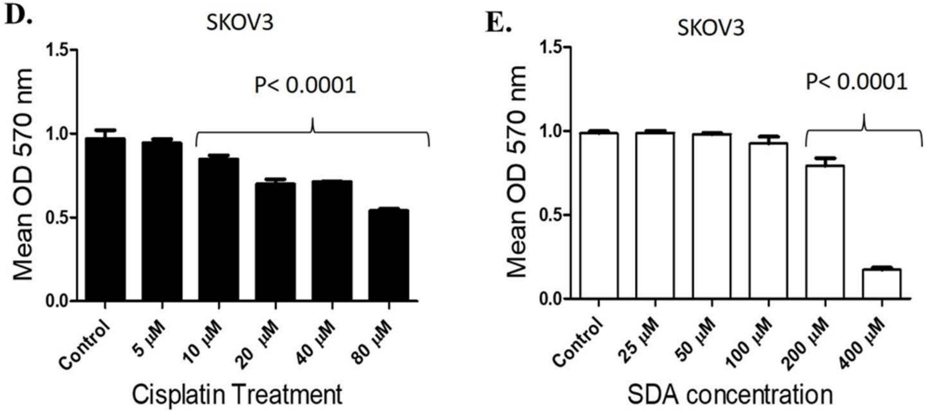
D.
SKOV3
Mean OD 570 nm
1.5
1.0
0.5
0.0
P< 0.0001
Control
5 µM
10 µM
20 µM
40 µM
80 µM
Cisplatin Treatment
E.
SKOV3
Mean OD 570 nm
1.5
1.0
0.5
0.0
P< 0.0001
Control
25 µM
50 µM
100 µM
200 µM
400 µM
SDA concentration

Supplementary Figure 1

Dose-response profiling of OVCAR5 and SKOV3 ovarian cancer cell lines to cisplatin and stearidonic acid (SDA).

(**A**) Cell line sensitivity curve depicting IC_{50} values across multiple ovarian cancer cell lines following cisplatin treatment. OVCAR5 (cisplatin-sensitive) and SKOV3 (cisplatin-resistant) lines are highlighted and annotated. https://www.cancerrxgene.org

(**B**) MTT viability assay in OVCAR5 cells treated with increasing concentrations of cisplatin (5–80 µM) for 72 hours shows a dose-dependent decrease in cell viability ($P < 0.0001$).

(**C**) OVCAR5 cells treated with SDA (25–400 µM) exhibit significant cytotoxicity at higher doses, with viability markedly reduced at 400 µM ($P = 0.0025$).

(**D**) SKOV3 cells show relative resistance to cisplatin, with only a gradual reduction in viability across increasing concentrations ($P < 0.0001$).

(E) SDA treatment in SKOV3 cells produces a similar dose-dependent response, with significant viability reduction at 400 µM ($P < 0.0001$).

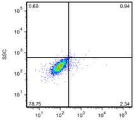

Kaiso expression
Skov-3 control stain with secondary Ab

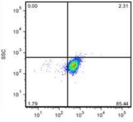

Kaiso expression
skov3-control stain

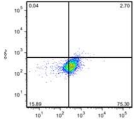

Kaiso expression
Sh-Scr

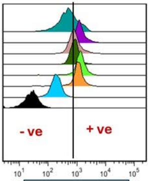

Kaiso Expression

Kaiso Expression Mean

	Sample Name	Mean , BluFL1
	o41818control unstain001.fcs	36.08
	o41818control stain with 2rv Ab002.fcs	255.87
	o41818control stain 003.fcs	1302.97
	o41818Scr2-F stain 005.fcs	1552.87
	o41818Scr2-G stain 007.fcs	1036.56
	o41818K2-U stain 009.fcs	1123.53
	o41818K2-E2best stain 011.fcs	1761.27
	o41818K2-a stain 013.fcs	744.41

Sh-kaiso stain is knocked down by approximately 57.17 % compared to Skov-3 control

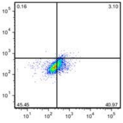

Kaiso expression
Sh-Kaiso

Supplementary Figure 2

Flow cytometry analysis confirms Kaiso knockdown in SKOV3 ovarian cancer cells. Representative dot plots (top row) and histogram overlays (bottom left) illustrate Kaiso expression in SKOV3 cell populations, including unstained control, secondary antibody control, scrambled shRNA (Sh-Scr), and Kaiso-targeted shRNA (Sh-Kaiso) conditions. Quantification of mean fluorescence intensity (MFI) in the BluFL1 channel (middle table) demonstrates a significant reduction in Kaiso expression in Sh-Kaiso–transduced cells (MFI = 744.41) compared to SKOV3 control-stained cells (MFI = 1,302.97), representing a ~57.17% decrease. These findings confirm efficient Kaiso knockdown at the protein level. Negative and positive staining thresholds were established using appropriate controls, as reflected in the histogram.

S1 table: Antibodies used for IHC and Western blotting:

#	Antibody target name	Name of Ab -cat#	Source
**1.**	**Kaiso**	Anti-Kaiso antibody [6F / 6F8] (ab12723)	Abcam
**2.**	**PXR**	PXR Antibody (G-11) sc-48403	Santa Cruz
**3.**	**NF-kB**	NFκB p65 (A-12): sc-514451	Santa Cruz
**4.**	**Her 2 (ERBB2)**	Anti-ErbB2 / HER2 antibody [EP1045Y]	Abcam
**5.**	**P-glycoprotein**	MDR1/ABCB1 Antibody (D-11): sc-58219	Santa Cruz
**6.**	**HIF-1A**	HIF1a Antibody (28b): sc-13515	Santa Cruz
